# Advances of Hydrogel-Based Bioprinting for Cartilage Tissue Engineering

**DOI:** 10.3389/fbioe.2021.746564

**Published:** 2021-09-29

**Authors:** Xue Han, Shuai Chang, Mingming Zhang, Xiangbing Bian, Chunlin Li, Dawei Li

**Affiliations:** ^1^ The Eighth Medical Center of PLA General Hospital, Beijing, China; ^2^ Department of Orthopedics, Peking University Third Hospital, Beijing, China; ^3^ Strategic Support Force Medical Center, Beijing, China; ^4^ The First Medical Center of Chinese PLA General Hospital, Beijing, China; ^5^ Senior Department of Orthopedics, The Fourth Medical Center of PLA General Hospital, Beijing, China

**Keywords:** bio-ink, bioprinting hydrogel, cartilage repair, polysaccharides, tissue engineering

## Abstract

Bioprinting has gained immense attention and achieved the revolutionized progress for application in the multifunctional tissue regeneration. On account of the precise structural fabrication and mimicking complexity, hydrogel-based bio-inks are widely adopted for cartilage tissue engineering. Although more and more researchers have reported a number of literatures in this field, many challenges that should be addressed for the development of three-dimensional (3D) bioprinting constructs still exist. Herein, this review is mainly focused on the introduction of various natural polymers and synthetic polymers in hydrogel-based bioprinted scaffolds, which are systematically discussed *via* emphasizing on the fabrication condition, mechanical property, biocompatibility, biodegradability, and biological performance for cartilage tissue repair. Further, this review describes the opportunities and challenges of this 3D bioprinting technique to construct complex bio-inks with adjustable mechanical and biological integrity, and meanwhile, the current possible solutions are also conducted for providing some suggestive ideas on developing more advanced bioprinting products from the bench to the clinic.

## Introduction

**FIGURE 1:**
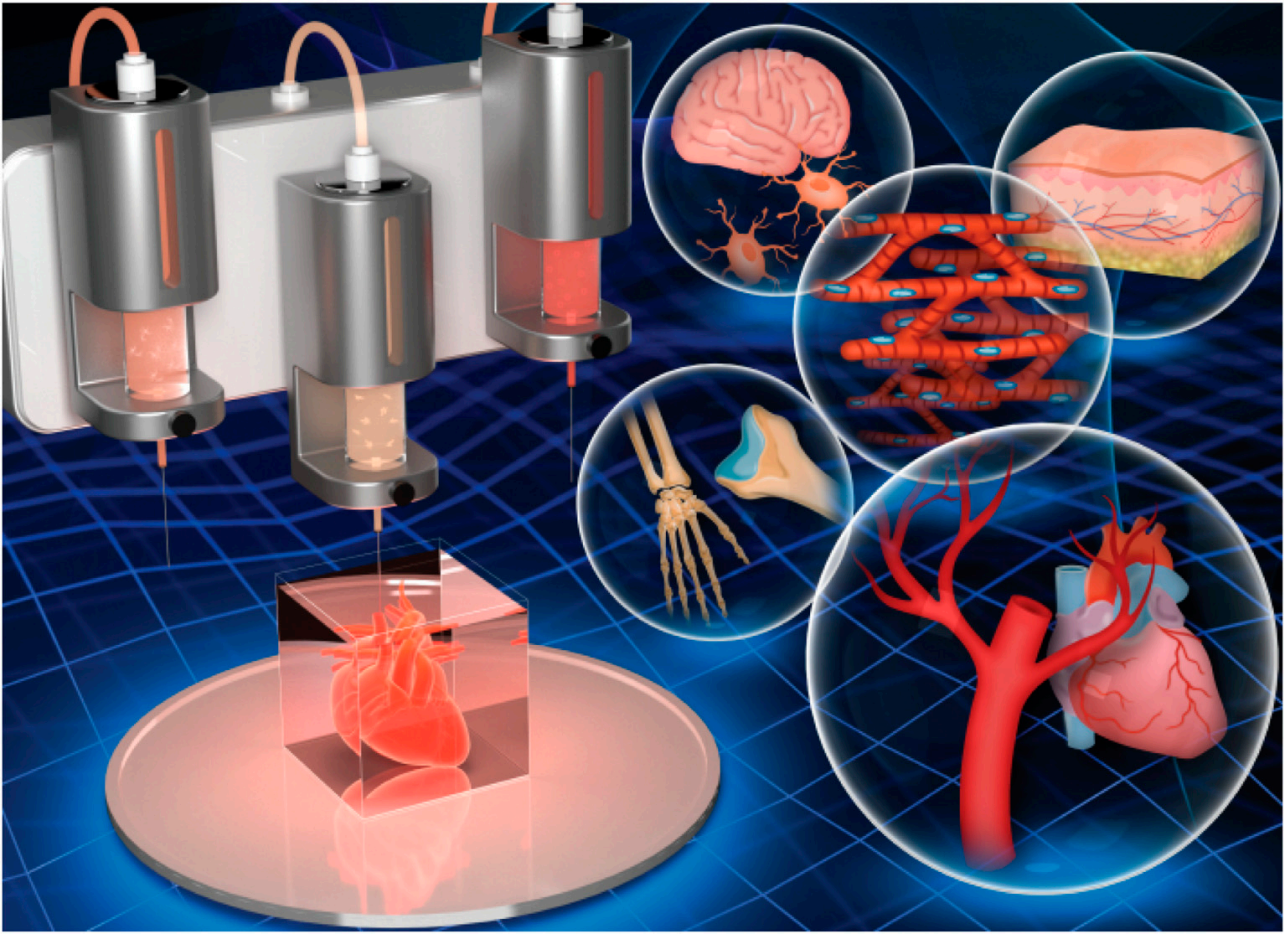
3D bioprinting of hydrogel constructs for tissue regeneration. Reproduced from Askari et al., (2021) with permission from Copyright 2021 Royal Society of Chemistry.

Bioprinting is defined as the spatial patterning of living cells and other biologics using computer-aided interlayer deposition to stack and assemble them to fabricate the living tissues and organs. As a novel 3D technology to print a variety of biological materials containing viable cells, bioprinting has received increasing attention worldwide and widely applied for broad-spectrum applications of regenerative medicines, tissue engineering and transplantation, pharmaceutical field, and cancer therapy (Ozbolat, 2015). Based on the precise deposition and administrative advantages, biomaterials, viable cells, drugs, and growth factors are concurrently deposited within a computer-aided layer-by-layer stacking pattern to obtain mimicking natural constructs, such as skin, bone, cartilage, lung, liver, and cardiac tissues (Figure 1) (Knowlton et al., 2016; Askari et al., 2021). Bioprinting technology always requires three main components: polymer solution, viable cells, and 3D printers. In general, bioprinting technology mainly relies on the advanced 3D bioprinters and designable bio-ink (polymer solution and viable cells), which can produce accurate tissue models with adjustable pore structures and diversified physicochemical features in a high-throughput fashion, thus facilitating the cocultivation of multiple or specific cells/organs and biological drugs/agents (Ozbolat and Yu, 2013; Park et al., 2016; You et al., 2016; Ji et al., 2019; Wang et al., 2019; Zhang et al., 2019).

Typical 3D-bioprinting scaffolds are stuffed with bioreactors with sufficient mechanical support and chemical stimuli to benefit the cell growth, which may be directly implanted to the infected site and kept in *in vitro* environment. Although modern bioprinting technology has a wide range of advantages, it currently faces some challenges that were closely related to how to tune the properties of the polymers for the preparation of the bio-inks. For example, the printed hydrogel is always too fragile to support the growth of hard tissues (e.g., bones and cartilage). In addition, the limited cell viability, adhesion, proliferation, growth, and differentiation on polymer-based scaffolds hinder the bioprinting of various tissues, and the specialized physiology and biological function of most cells can be severely impaired *in vivo* (Hong et al., 2015). For example, the selected polymer solution requires low viscosity, stiffness, and cross-linking to promote cell migration, nutrient diffusion, and tissue formation. In contrast, these polymers are also required with high viscous and stiff to fulfill dimensional accuracy and mechanical support. These contradictory requirements demonstrate a challenge balance between the architectural topology, physico-mechanical property, and biological activity to ensure the high cellular viability of the bioprinting materials. Moreover, co-printing various cells always needs varied bio-inks with more complicated printing process, and these prepared bio-inks have limited shelf-life and face storage difficulties (Murphy and Atala, 2014). Together, it still faces great challenge in bioprinting of natural-like full-scale organs.

Hydrogel with 3D cross-linked networks is a kind of essential components of bio-ink, which can directly contact with cells, keep cell activity, and support their adhesion, growth, and proliferation. On account of the adjustable physico-mechanical properties, high water content, and advanced biodegradability, hydrogel is recognized as an ideal bio-ink for cell bioprinting. Hydrogels have the advantages of biocompatibility, biodegradation, facile injection growth under specific conditions, convenient transportation of nutrients during development, and easy to be used in different places. However, poor mechanics greatly impair its tissue regeneration applications like cartilage, bone, and other organs. Although there are many published reports about the various methods to improve the mechanical performance *via* physical or chemical cross-linking with other materials, nanoparticles, and inorganic agents, these traditional hydrogels do not have satisfying capacities to provide uniform porous structures to allow sufficient exchange and filtration of nutrients and offering suitable ECMs to support cell growth (Grande et al., 1997). Therefore, an ideal hydrogel should take both mechanical and biological properties into account for high-resolution printing and cell viability, which require designable structures, highly porous networks, biocompatibility, biodegradability, favorable mechanics, suitable viscosity, and excellent printability.

Bioprinting hydrogels are categorized into two natural and synthetic polymer–based hydrogels. Natural polymers like hyaluronic acid, gelatin, chitosan, and collagen have good hydrophilicity, biocompatibility, safety, and biodegradability, but the poor mechanical properties limit their bio-applications. Although synthetic biomaterials possess good mechanics, adjustability, and mechanical stability, their hydrophilicity and cell compatibility are lower (Tan and Marra, 2010; Gasperini et al., 2014; Tang et al., 2020). Therefore, in order to create a matrix with favorable cell compatibility and mechanical strength, this current review describes the potential of various polymer-based hydrogel bioprinting with their unique characteristics and tunable properties, which can promote cell adherence, proliferation, and migration on bioprinting scaffolds for cartilage repair. It is also focused on the current challenges on bioprinting technology for broad-spectrum practical applications in tissue engineering and regenerative medicine. This review can systemically describe the relationship between polymer sciences and bioprinting technology in applications of cartilage regeneration.

## Bioprinting Hydrogel Constructs

Although 3D bioprinting technology is recognized as an effective method for cartilage repair, scaffold design and preparation still face some challenges due to the biological and mechanical performances of bio-inks (Derby, 2012; Chia and Wu, 2015; Kumar et al., 2016). An ideal bio-ink should meet the integrated printability, cell compatibility, and mechanical property for tissue engineering applications, but there is a trade-off between cell culture and the ideal 3D printing bio-ink. Therefore, many bio-inks lack suitable printability and shape fidelity, insufficient biodegradability and biological activity, and weak mechanical strength (Mandrycky et al., 2016).

Among various bio-inks, hydrogels have gained most popularity because of their hydrophilic polymer chains, high water absorbability, adjustable topologies, designable size, biological property, and cell/drug carriers in tissue engineering and drug delivery. For example, cell, nutrients, drugs, and growth factors can be directly loaded into the hydrogels *in situ* to recapitulate native tissue microenvironment, which can provide comfortable conditions to benefit the cell adhesion, migration, growth, proliferation, and differentiation. Therefore, hydrogel-based systems have been a prime candidate in cartilage tissue engineering using 3D bioprinting techniques (Piras et al., 2017; Chimene et al., 2020). By means of advanced physical means (blending with functional nanoparticles) and chemical methods, physicochemical capacities of hydrogels can be easily tailored, but the complexity of the biological tissue–like porous network, bionic structure, beneficial cell compatibility, mimicking ECM environment, and biomechanical support is difficult to be replicated completely for cellular activities in biomedical applications because most of the biological processes are always heterogeneous in nature. Thereby, development of various innovative preparation technologies not only satisfies the dynamic adjustment for gradual shape change in a predetermined path but also controls spatial heterogeneity to meet the needs of organization integration, local cell behavior, and cell–substance interaction (Daly et al., 2016; Donderwinkel et al., 2017; Ligon et al., 2017; Zheng et al., 2018; Ashammakhi et al., 2019).

The outstanding mechanics (e.g., cross-linking degree and stiffness) of traditional bio-inks require high density that is not benefited for the cell growth and matrix deposition. Conversely, low density of bio-inks can reduce their shape fidelity, making it difficult to retain shape after post-printing (Cheng et al., 2008). This contradiction hinders to achieve ideal cell culture. Therefore, new bio-inks are required to design with simultaneous biocompatibility and printability *via* the precise control of physical, chemical, and biological properties. There are a number of performance metrics that need to be met, such as viscoelasticity, viscosity, gelation kinetics, cell migration, growth and proliferation, porous structures, and biodegradation. In order to solve the trade-off between cell viability and the feasible printability, photo cross-linking bio-inks have been proposed and developed *via* the simple *in situ* cross-linking strategy, which is directly cured by photo-permeable capillary before the deposition (Daly et al., 2017; Piras et al., 2017; Galarraga et al., 2019). However, clogging and the stability are some common defects for preparing the structurally integrated bio-inks. Therefore, many developed strategies are widely applied to repair the focal cartilage defects based on the polymer-based bioprinting hydrogels.

## Polymer-Based Bioprinting Hydrogels for Cartilage Tissue Engineering

Cartilaginous tissue possesses a hydrated heterogeneous and avascular structure with specific characterizations of low friction, wear-resistant, and load-bearing surfaces for effective joint protection and movement (Yan et al., 2020). Although many conventional surgical treatments have been proposed for chondral injuries, few clinical studies have shown the reliable regeneration of normal hyaline cartilage due to its poor self-healing capacity originating from the insufficient supply for defected sites. In addition to the difficult structural similarity to the native tissue, its functional regeneration of articular cartilage like the different cell morphologies, mimicking ECM environment, cell activity, and growth, was full of challenges (Xu et al., 2020). Moreover, scaling up bioengineered transplants to match native structures is also challenging because the diffusion of nutrients and metabolites may be affected. Therefore, the advent of 3D bioprinting technology provides the precise architecture and biological multifunction to the native tissues, which have attained prominent progress on fabrication of many cartilage-like hydrogel constructs for cartilage tissue engineering. Hydrogel constructs mainly consist of natural and synthetic polymer-based or combination of both (Fan et al., 2020a; Li et al., 2020a; Bao et al., 2020; Yu et al., 2020; Zhu et al., 2021), which are used and selected for the construction of 3D printable bio-inks for cartilage tissue regeneration.

### Natural Polymer-Based Bioprinting Hydrogels for Cartilage Tissue Engineering

Natural polymers mainly include hyaluronic acid, gelatin, cellulose, alginate, chitosan, starch, silk, and fibroin, which are important elements for providing ECM environments to cells and widely applied for bioprinting hydrogels in tissue regeneration.

#### Hyaluronic Acid

**FIGURE 2:**
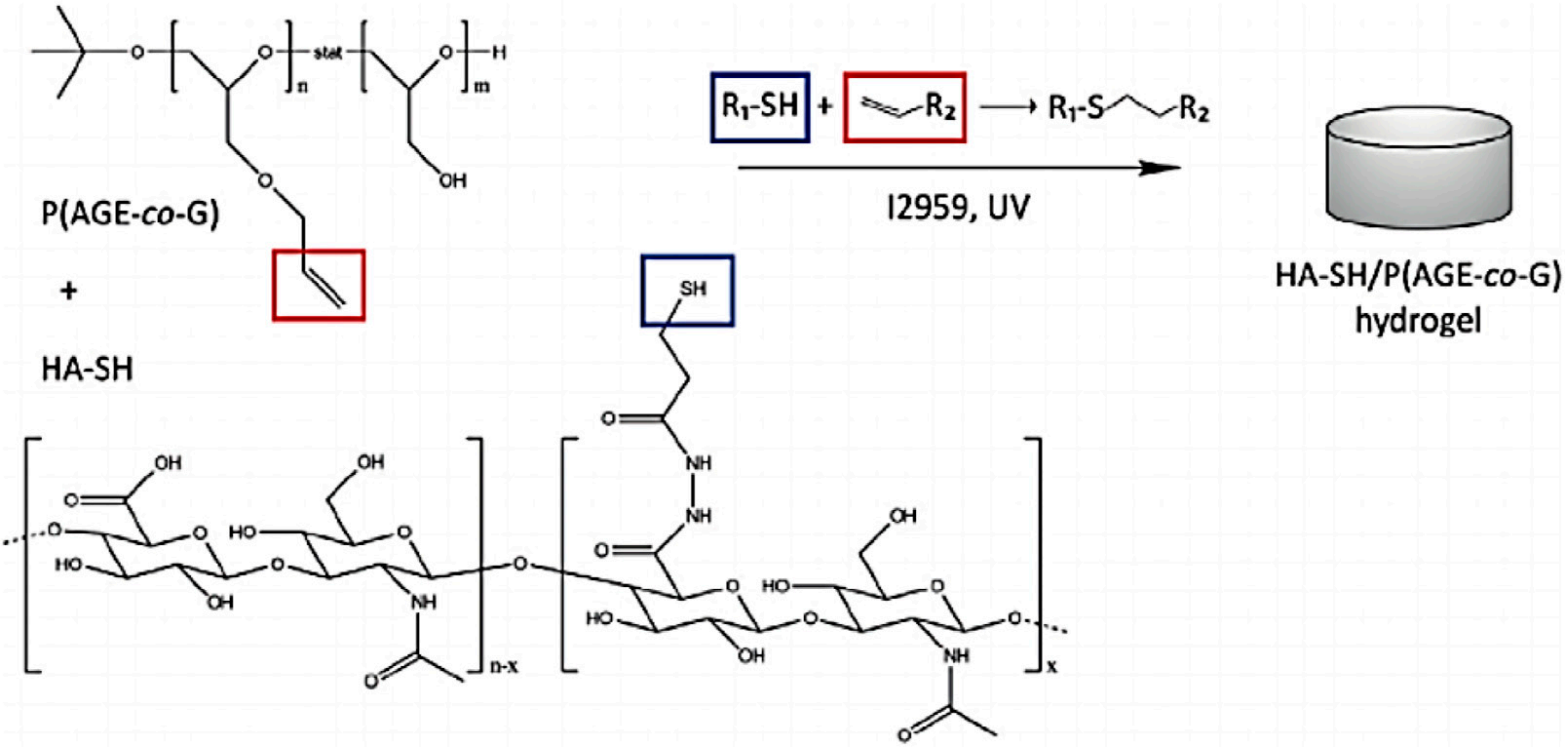
Chemical structure and cross-linking mechanism of hydrogels. Reproduced from Hauptstein et al., (2020) with permission from Copyright 2020 Wiley.

Hyaluronic acid (HA) is a non-sulfated linear polysaccharide with outstanding biocompatibility, biodegradability, and high viscoelasticity, which has been a promising ECM material for the construction of bio-inks using the bioprinting technology (Pescosolido et al., 2011). However, its high hydrophilicity leads to the weak stability of bioprinting hydrogel constructs. To address this dilemma, many strategies have been developed by physical blending and chemical cross-linking methods with other functional moieties. Nedunchezian et al. developed a kind of HA-based bioprinting HA–biotin–streptavidin (HBS) hydrogels *via* chemical cross-linking modification and noncovalent bonding interactions for the cartilage repair (Nedunchezian et al., 2021). These hydrogels were further treated with sodium alginate and immersed into CaCl_2_ solution to obtain a stable 3D HBSAC (HBSA + Ca^2+^) hydrogel scaffold *via* ion cross-linking. The physicochemical characteristics of the hybrid bio-ink had been testified with good printability and structural integrity, which was benefited for the chondrogenic differentiation both *in vitro* and *in vivo*. Hauptstein et al. prepared a HA-based bio-ink composition for cartilage regeneration (Figure 2). By means of the UV light, thiolated HA and allyl-modified poly (glycidol) were cross-linked to form the hydrogels (Hauptstein et al., 2020). Then unmodified high molecular weight of HA was further blended to form composite hydrogel constructs, which promoted the chondrogenic differentiation of human mesenchymal stromal cells with good biological and mechanical properties.

#### Gelatin

Gelatin is derived from the collage *via* hydrolytic degradation and is able to possess outstanding biocompatibility, cytocompatibility, degradability, low melting point, and favorable thermal stability, which has made great progress for the tissue engineering scaffolds (Li et al., 2018). As a bioprinting hydrogel construct, gelatin has the amino acid composition and sequence within its backbone, and therefore displays the unique amphoteric capacity for bio-ink application. Huang et al. prepared the gelatin/hydroxyapatite hybrid materials through the microextrusion 3D bioprinting technique and enzymatic cross-linking method to obtain the hydrogel scaffolds, which could play an important role in improving cell adhesion, proliferation, and growth to promote the chondrogenic differentiation (Huang et al., 2021). *In vivo* results showed this implanted cell-laden bioprinting scaffold exhibited effective articular cartilage regeneration using a pig model. On account of the precise architecture and suitable porosity by 3D printing technique, these biocompatible scaffolds demonstrated the outstanding chondrogenic differentiation behaviors and potential treatment for the cartilage injury.

Yang et al. developed a 3D-bioprinted difunctional scaffold for articular cartilage regeneration. By means of the photo-crosslinking method, the aptamer and TGF-β3 were encapsulated to form the functional GelMA bio-ink, which could recruit MSCs and promote cell chondrogenic differentiation (Yang et al., 2021a). This bio-ink was further co-printed with PCL for improving the mechanical property. Therefore, this 3D-bioprinted difunctional constructs could not only recruit the MSCs and offer the bionic microenvironment for cell growth and chondrogenesis but also provide the powerful mechanical support for neotissue maturation and cartilage repair using a rabbit model.

**FIGURE 3:**
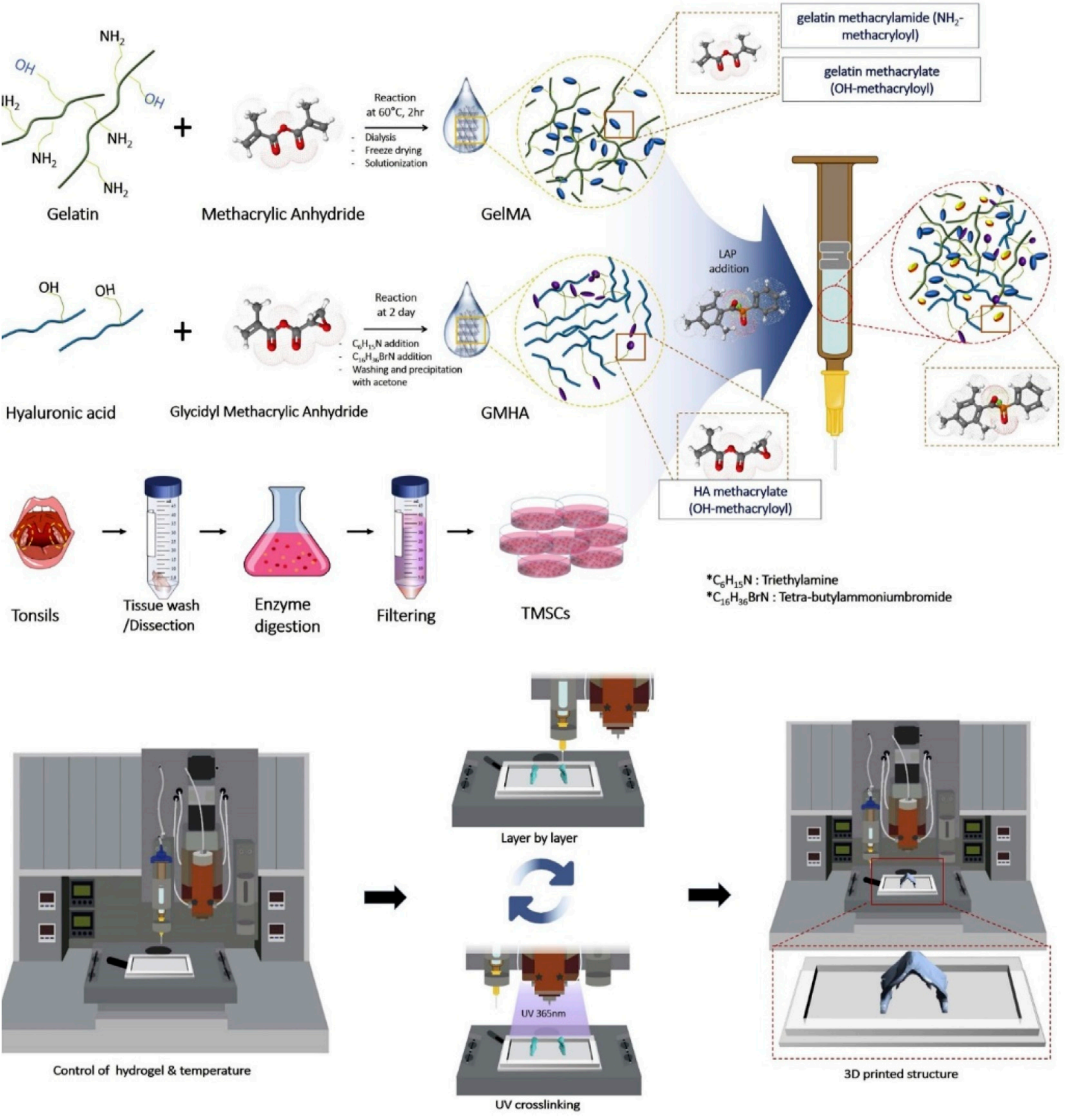
Scheme of the preparation of GelMA/GMHA bio-ink using the 3D bioprinting technique. Reproduced from Lee et al., (2020) with permission from Copyright 2020 Elsevier.

Methacrylate gelatin (GelMA) is prepared by the modification of gelatin with the methacrylate groups, which possess similar composition to the natural ECM to facilitate cell adhesion and growth. By means of the photo-crosslinked strategy, these biocompatible GelMA hydrogels were widely used for 3D bioprinting hydrogels with good biological and adjustable physicochemical properties (Lam et al., 2019). In recent years, these GelMA hydrogels were generally combined with other biomaterials to acquire the multifunction for satisfying tissue engineering. For example, Luo et al. demonstrated a cell-laden GelMA-based bio-ink with excellent cell activity and chondrogenesis by the photo-crosslinking method (Luo et al., 2020). By means of precise extrusion bioprinting technology, the printed GelMA hydrogel was obtained with the continuous microfibers and stabilized by the photo-crosslinking method. This bio-ink could express the chondrogenic gene by the immunofluorescence after culturing the constructs into the chondrogenic medium *in vitro*, and *in vivo* results showed that these cell-laden bio-ink possessed good printability, structural integration, and high cell viability, which could successfully promote the effects of cartilage regeneration. Lam et al. used a stereolithographic bioprinting approach to achieve a tissue-engineered cartilage bio-ink with the main components of biocompatible GelMA and methacrylated hyaluronic acid (Lam et al., 2019). These bioprinting constructs possessed good stability, chondrogenic viability, and differentiation, which could obviously support cartilage ECM formation and enhance cartilage-typical zonal segmentation for effective treatment of cartilage defects. Lee et al. prepared a hybrid bio-ink comprising GelMA and glycidyl-methacrylated HA (GMHA) for the cartilage repair (Figure 3). After optimizing the ratios, 7% GelMA and 5% GMHA bio-ink was selected with good mechanics, rheology, and printability. *In vitro* and *in vivo* results verified the important roles in offering the suitable microenvironment for stem cell chondrogenesis and cartilage tissue regeneration (Lee et al., 2020).

#### Cellulose

**FIGURE 4:**
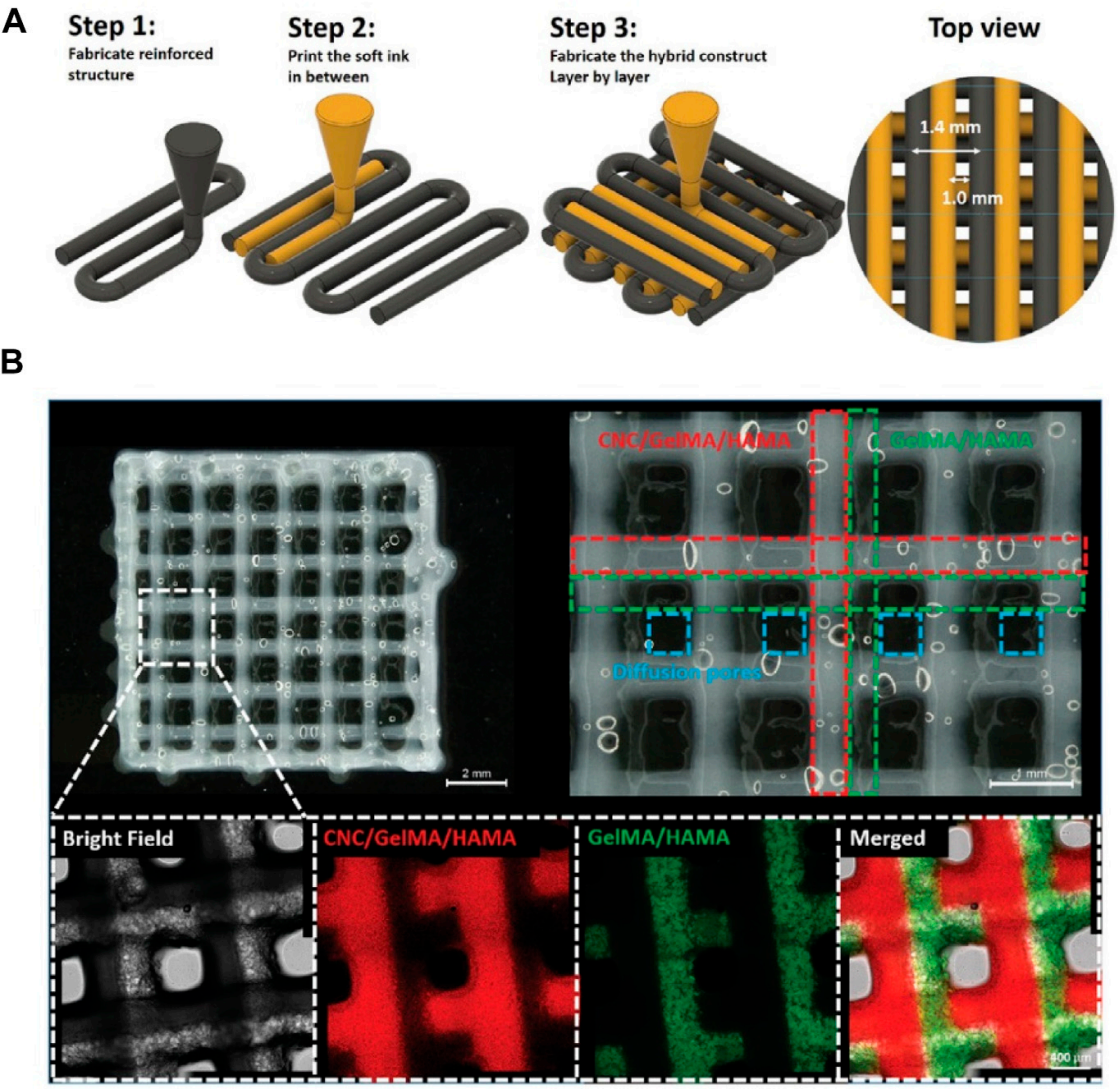
**(A)** Illustration of the hybrid printing procedure. **(B)** Optical and confocal microscopic observation of the hybrid printed construct. The resulting holes are defined by blue dashed lines. The fluorescence in confocal images is from fluorescence-labeled GelMA (red: rhodamine-labeled GelMA; Green: FITC-marked GelMA) in CNC-enhanced inks or GelMA/HAMA inks. Reproduced from Fan et al., (2020b) with permission from Copyright 2020 Wiley.

Cellulose comprises glucose units by the β-(1–4)-glycosidic linkages, which is one of the most available natural polymers (Ul-Islam et al., 2014; Ul-Islam et al., 2013; Yin et al., 2017). On account of its advantages on hydrophilicity, water-locking ability, biocompatibility, and biodegradability, cellulose has been greatly applied in the biomedical application. Cellulose is classified with micro-fibrillated, nano-crystalline cellulose and bacterial nanocellulose (BNC). The BNC possesses similar size of collagen fibrils that is widely used for constructing cartilage tissue engineering scaffolds. It is reported that nanocellulose-based bio-inks can be used as hydrogels for 3D bio-printing of living cells. For example, Fan et al. presented a hybrid printing platform of two hybrid hydrogel bio-inks for achieving the simple fabrication and seamless structural integration (Figure 4) (Fan et al., 2020b), wherein cellulose nanocrystals and GelMA/HA were photo-crosslinked to improve mechanical properties and cell growth environment. These hybrid bioprinting scaffolds could encapsulate the chondrogenic cell using two optimized inks with the layer-by-layer method, and the cell viability was basically unaffected during the printing procedure, which suggested its valuable applications for cartilage regeneration.

Apelgren et al. developed a cartilage-specific bio-ink *via* the aqueous counter collision strategy, which could prolong the fibrils and induce the negative charges (Apelgren et al., 2019). After encapsulating the human nasal chondrocytes into the scaffolds, these cell-laden constructs presented good printability, structural stability, and integrity, thus demonstrating the outstanding chondrocyte proliferation using the nude mice at 2 months. *In vivo* results showed that a full-thickness skin graft was integrated on these cell-laden bio-inks with potential clinical translation for cartilage repair.

#### Alginate

**FIGURE 5:**
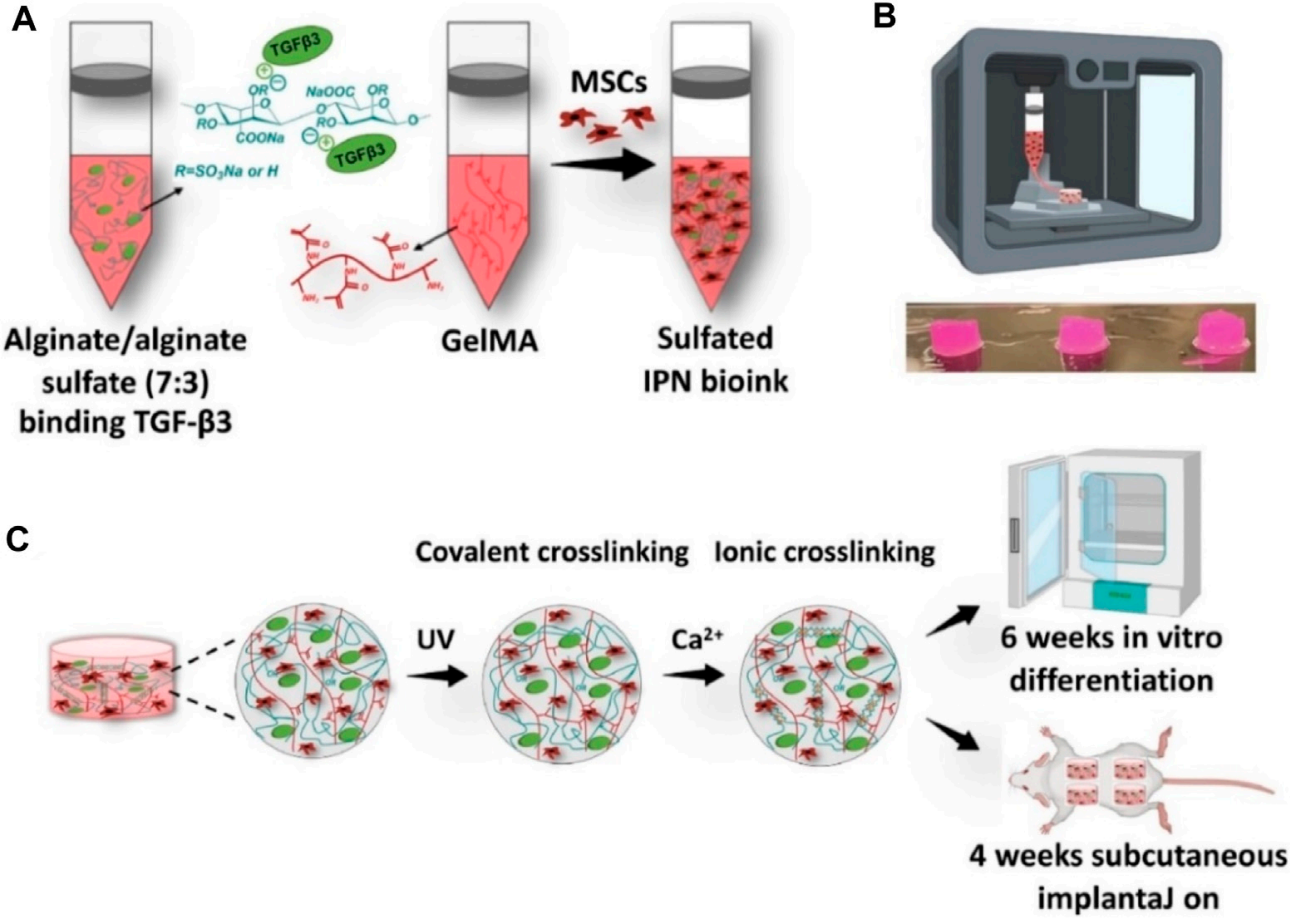
Schematic representation of alginate/alginate sulfate-GelMA IPN bio-ink and bioprinting constructs using the ionic cross-linking strategy. **(A)** Preparation of bio-ink. **(B)** Image of 3D-bioprinting constructs. **(C)** Cross-linking processes. Reproduced from Wang et al., (2021) with permission from Copyright 2021 Elsevier.

Alginate or sodium alginate (raw form), as an anionic polysaccharide from seaweed source, consists of mannuronic acid and guluronic acid units (Ahn et al., 2012). With a similar structure to the native ECM, alginate has good biocompatibility, high viscosity, and facile gelation for the development of promising bioprinting constructs. However, due to its insufficient ability for cell adhesion and proliferation, alginate is acquired by blending and mixing some other compatible components or cell-adhesion moieties. Based on this, cell-laden bioprinting alginate hydrogels are designed and prepared in recent years. For example, Yu et al. developed a scaffold-free strategy on fabrication of an engineered scaffold-free alginate stretchable tissue strand as a novel robot-assisted bio-ink, which achieved the construction of an 8-cm long tissue strand with the rapid fusion property, thereby avoiding the requirement for solid supports or molds (Yu et al., 2016). Moreover, Kim et al. developed a multilayered composite scaffold through coating an alginate-based bio-ink on the PCL surface, which possessed the powerful mechanics and outstanding cell viability for tissue repair (Kim et al., 2016). Wang et al. prepared a functional alginate–gelatin bio-ink with the interpenetrating network (IPN) for cartilage repair. Embedding of alginate sulfate had a limited effect on the viscosity and shear-thinning properties, which could enable the high-fidelity bioprinting and support the MSC viability (Figure 5) (Wang et al., 2021). The rigidity of the bioprinting constructs was greatly improved compared to that of individual alginate or GelMA bio-ink. In addition, due to high affinity on the heparin-binding growth factors, it possessed a sustained release behavior of TGF-β3 and promoted the chondrogenesis both *in vitro* and *in vivo* with high level of cartilage-specific extracellular matrix deposition.

#### Chitosan

**FIGURE 6:**
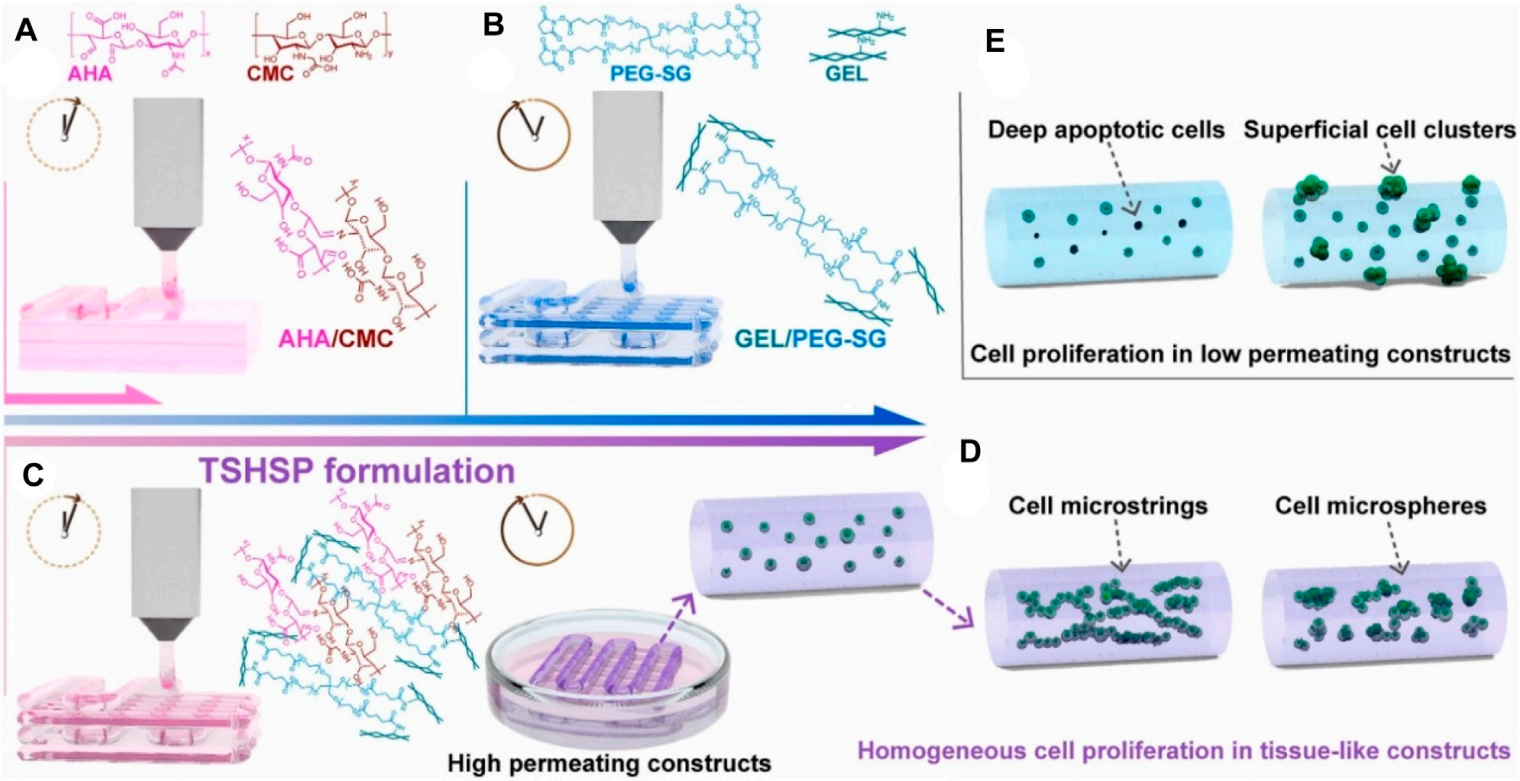
Schematic representation of bioprinting TSHSP hydrogel constructs from AHA/CMC and GEL/PEG-SG. **(A)** Microstructure fusion of constructs. **(B)** Time-consuming pre-printing preparation. **(C)** TSHSP formulation by the complementarity of AHA/CMC and GEL/PEG-SG. **(D)** Uniform proliferation pattern of cells in tissue-like constructs. **(E)** Proliferation pattern of cells in low permeating constructs. Reproduced from Chen et al., (2021) with permission from Copyright 2021 Elsevier.

Chitosan is a linear amino-polysaccharide with the backbone of β-(1–4)-linked D-glucosamine, which possesses favorable biocompatibility, biodegradability, high viscosity, and unique antibacterial property for bio-applications (Yang et al., 2018; Li et al., 2020b; Tang et al., 2020; Yang et al., 2021b; Yang et al., 2021c). However, its poor solubility in neutral pH requires the manufacturing process in acidic condition for bio-ink preparation, which greatly limits cell viability and proliferation, especially for the direct bioprinting. To overcome this problem, various alternative strategies have been developed. For example, He et al. prepared a carboxymethyl chitosan-based 3D bioprinting bio-ink for the cartilage repair. Based on the physical crosslinking between the carboxyl groups and calcium, the mechanical properties of hydrogel scaffold were improved. Moreover, *in vitro* and *in vivo* experiments showed the favorable cell viability, attachment and proliferation, and obvious chondrogenic expression to verify the cartilage regeneration (He et al., 2020). Chen et al. synthesized a structure-supporting, self-healing, and high permeating hydrogel bio-ink, which was composed of aldehyde HA (AHA)/N-carboxymethyl chitosan (CMC) and gelatin (Gel)/4-arm poly (ethylene glycol) succinimidyl glutarate (PEG-SG) (Chen et al., 2021). The obtained hydrogel bio-inks possessed good printability, self-healing capacity, and high permeability, thus achieving long-term cell viability, attachment, and growth for biological application (Figure 6).

#### Silk

**FIGURE 7:**
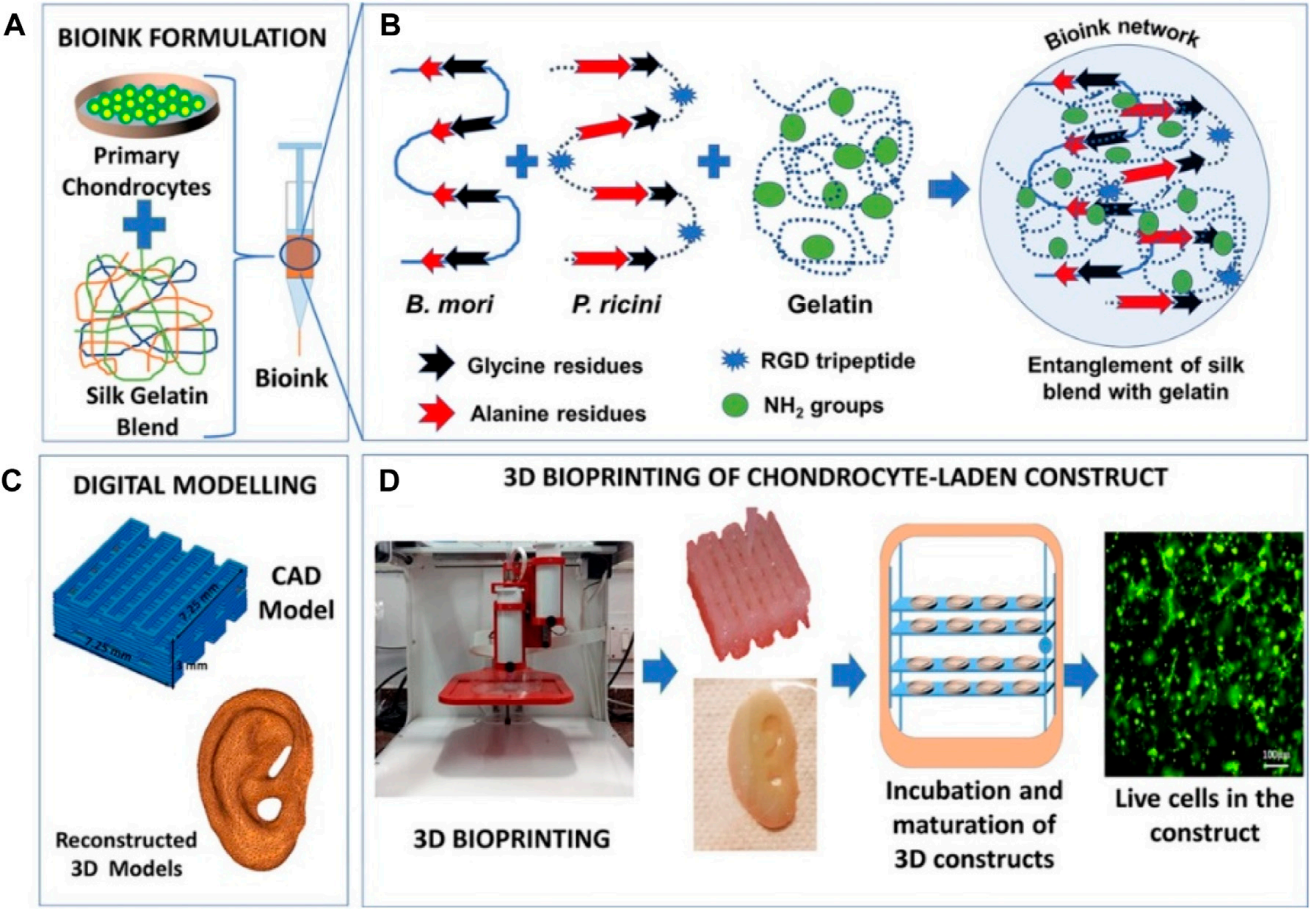
Schematic representation of **(A)** bio-ink formulation, **(B)** polymer entanglement and interaction, **(C)** digital modeling, and **(D)** 3D bioprinting and maturation of cell-laden construct. Reproduced from Singh et al., (2019) with permission from Copyright 2019 American Chemical Society.

Silk is a natural protein polymer found in the glands of arthropods, and silk fibers were extracted from silk with the main components of silk fibroin and sericin proteins, which has vastly progressed in tissue engineering and drug delivery applications (Lamboni et al., 2015; Gregory et al., 2016). However, this progress has been challenged by the difficulty of developing bioprinting materials, because most bio-inks require toxic chemical cross-linking. To overcome this problem, Singh et al. developed silk fibroin blends with gelatin through the polymeric chain entanglements and physical cross-linking interactions, which were printed as bio-inks with suitable swelling behavior, optimal rheology, and supportive structure (Figure 7) (Singh et al., 2019). By increasing the content of sulfated glycosaminoglycan and collagen, this bioprinting construct allowed the chondrocytes growth and proliferation to generate the cartilage extracellular matrix and upregulate the chondrogenic gene expression, which indicated this cross-linker–free silk-gelatin may be explored as potential candidates in cartilage regeneration.

Ni et al. used the silk fibroin (SF) and hydroxypropyl methyl cellulose (HPMC) to construct a novel cell-laden double network hydrogel through 3D bioprinting technology for cartilage repair (Ni et al., 2020). SF possessed a high β-sheep structure as the first rigid and brittle network and HPMC was acted as the second soft network; in this case, this hydrogel exhibited high strength and remarkable compressive reproducibility, which could support the cell growth and proliferation. After encapsulation of bone marrow mesenchymal stem cell (BMSC), this SF-based bio-inks also expressed the outstanding biochemical property for cartilage tissue regeneration.

#### Protein

Fibrin is a fibrous protein and component of native ECM formed by the interaction between protease thrombin and fibrinogen, which can combine with the platelets from the hemostatic clots for blood coagulation and has been widely used for cartilage engineering tissues due to its biocompatibility, biodegradability, and nanofibrous structural properties. The presence of cell adhesion motif and cell compatibility allows fibrin to benefit in biological printing applications. However, fibrin soft and fragile characteristics lead to the poor structural integrity and cannot maintain shape fidelity for 3D biofabrication and bioprinting constructs. Therefore, it is committed to overcoming this problem by combining fibrin with various polymers. For example, Ning et al. prepared a Schwann cell-laden scaffold using the bioprinting technique with the main components of RGD modified alginate, hyaluronic acid, and fibrin, which exhibited outstanding machinability and biological properties for the tissue regeneration (Ning et al., 2019). Honarvar et al. prepared a kind of polycaprolactone (PCL) scaffolds by coating with fibrin and cartilage acellular ECM using 3D-printing methods. After encapsulating the adipose-derived stem cells (ADSCs) into fibrin/ECM hydrogel and seeding onto the PCL scaffolds, this 3D-printed constructs possessed higher compressive properties and water uptake for benefiting the cell growth, which indicated the important roles in cartilage tissue engineering (Honarvar et al., 2020).

In addition, soy protein is an important natural polymer from shelled and degreased soybeans, which accounts for 6% of the total polymer distribution used in bioprinting applications. The structure and physiological characteristics of soybean protein can be adjusted by various processing. Its improved thermoplastic properties and high biocompatibility allow it to be used for the development of porous scaffolds and tissue engineering applications (Manan et al., 2017).

### Synthetic Polymer–Based Bioprinting for Cartilage Tissue Engineering

The emerging 3D printing technology is the result of the combination of biological materials and related technologies such as biology, chemistry, physics, mechanics, and medicine. Distinct from natural polymers with similar ECM components, synthetic polymers are generally required to combine with other natural polymers to tailor the degradability and improve the biological property through reasonable physical–chemical methods for bioprinting application (Li et al., 2016; Wang et al., 2016; Wang et al., 2018). Synthetic polymers play a key role in supporting cellular and biomolecular (or bioactive) activity before, during, and after the 3D printing process. In particular, biodegradable synthetic polymers have excellent mechanical properties, tunable chemical structure, nontoxic degradation products, and controllable degradation rate, making them the first choice for the manufacture of cartilage tissue engineering.

#### Poly(ethylene Glycol)

**FIGURE 8:**
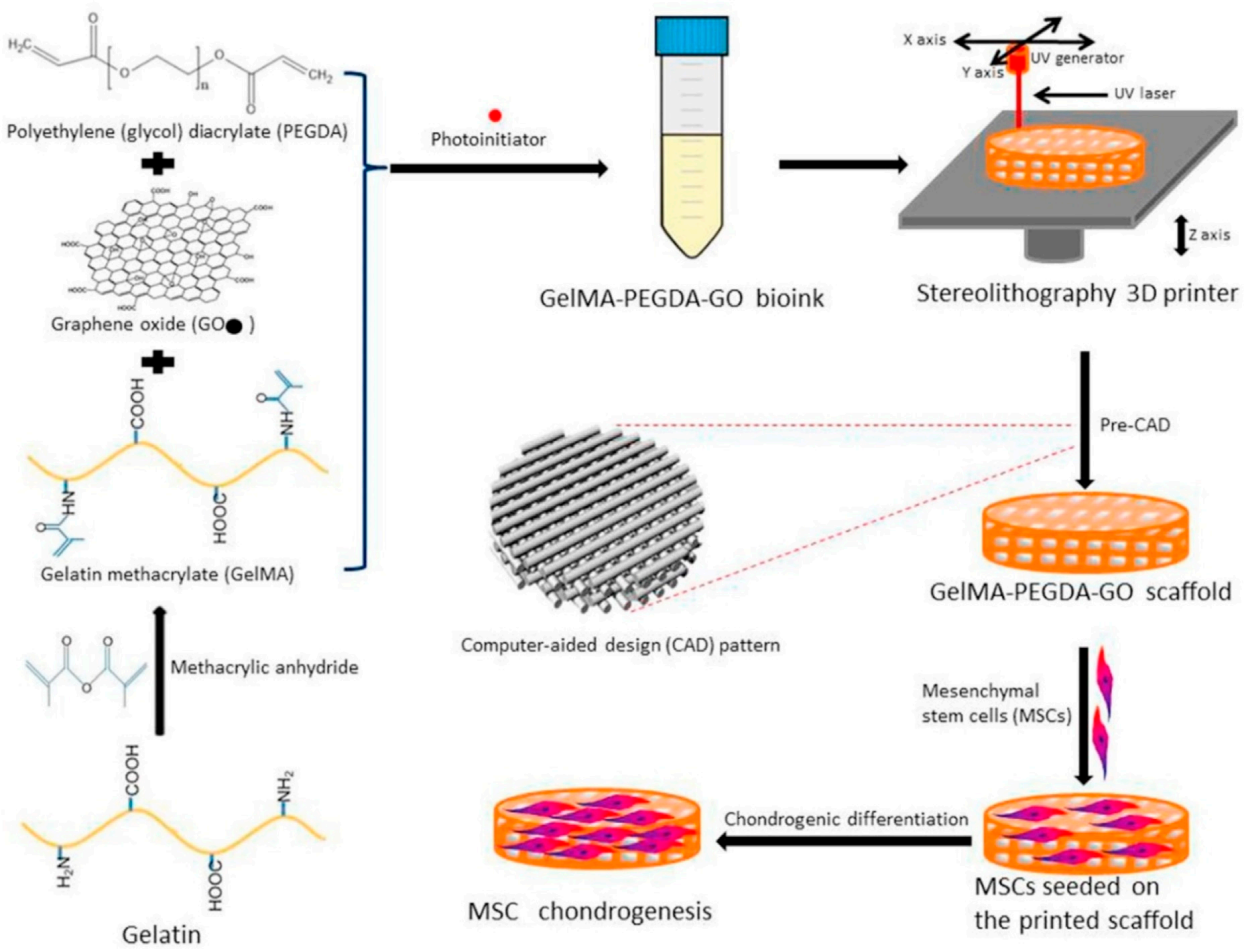
Schematic preparation of bioprinting scaffold to promote chondrogenic differentiation. Reproduced from Zhou et al., (2017) with permission from Copyright 2017 Royal Society of Chemistry.

PEG was a promising material for bioprinting hydrogel due to its good biocompatibility, high solubility, and easy modification. In general, poly (ethylene glycol) diacrylate (PEGDA) was applied for construction of hydrogel bio-ink through UV-induced polymerization. Daniele et al. prepared an interpenetrating network with the main component of GelMA and PEG, which could satisfy the requirement of cell attachment and growth within this composite bioprinting constructs (Daniele et al., 2014). Mouser et al. designed a bioprinting construct with suitable biological and mechanical properties, which consisted of methacrylated hyaluronic acid (HAMA) and methacrylated poly [N-(2-hydroxypropyl) methacrylamide mono/dilactate] (pHPMAlac)/PEG triblock copolymers (Mouser et al., 2017). After co-printing with PCL and encapsulation of chondrocytes, the cell-laden bio-ink displayed the tunable internal architectures, improved the mechanical properties, and promoted the glycosaminoglycan (GAG) and collagen type II contents, which were found to be optimal for cartilage-like tissue formation. Zhou et al. developed 3D bioprinting scaffolds with the components of graphene oxide (GO)-GelMA and PEGDA, which could promote chondrogenic differentiation with the improving glycosaminoglycan and collagen levels (Figure 8) (Zhou et al., 2017).

#### Poly (L-Lactide-Co-Glycolide)

PLGA which consisted of a copolymer of lactide and glycolide had received significant attentions for bio-ink application due to its adjustable structures, good biocompatibility, and biodegradability (Wang et al., 2022). Gottardi et al. prepared a biphasic bioprinting PLGA scaffold with embedding adult human mesenchymal stem cells for chondrogenic regeneration (Gottardi et al., 2021). The *in vitro* assay showed that constructs cultured in a two-fluid bioreactor differentiated locally into tendinocytes or chondrocytes according to exposure to the appropriate differentiation medium after culturing for 7, 14, and 21 days. The cartilage matrix marker, collage II, and tendon-specific marker were significantly unregulated in the cartilaginous and tendinous portions at 21 days, indicating the favorable dual fluidic system for fabrication of the tendon enthesis-like biphasic constructs.

#### Poly(ε-Caprolactone)

PCL was a kind of biodegradable synthetic polyester for wide applications in tissue regeneration because of its outstanding thermoplastic behavior, high mechanics, suitable biodegradability, and easy processability (Zhou et al., 2020). Koo et al. developed a 3D bioprinting cell-laden collagen and hybrid constructs using PCL plates for articular cartilage tissue regeneration, which possessed optimal porous mesh structures and high mechanical properties to support the chondrocyte viability within the bio-inks *in vivo* (Koo et al., 2018). Visser et al. printed a PCL-based hydrogel scaffold with a highly porous microfiber through an electro-spun technology. This construct with good stiffness and elasticity could support the chondrocyte viability and growth (Visser et al., 2013). Kim et al. developed a multilayered PCL/alginate bio-ink constructs using the cell-printing strategy (Kim et al., 2016). The laden cells could display a substantially more developed cytoskeleton with a homogenous distribution within the constructs, and these cell-laden bio-inks exhibited the outstanding osteogenic activity.

#### Poly(ε-Caprolactone)

**FIGURE 9:**
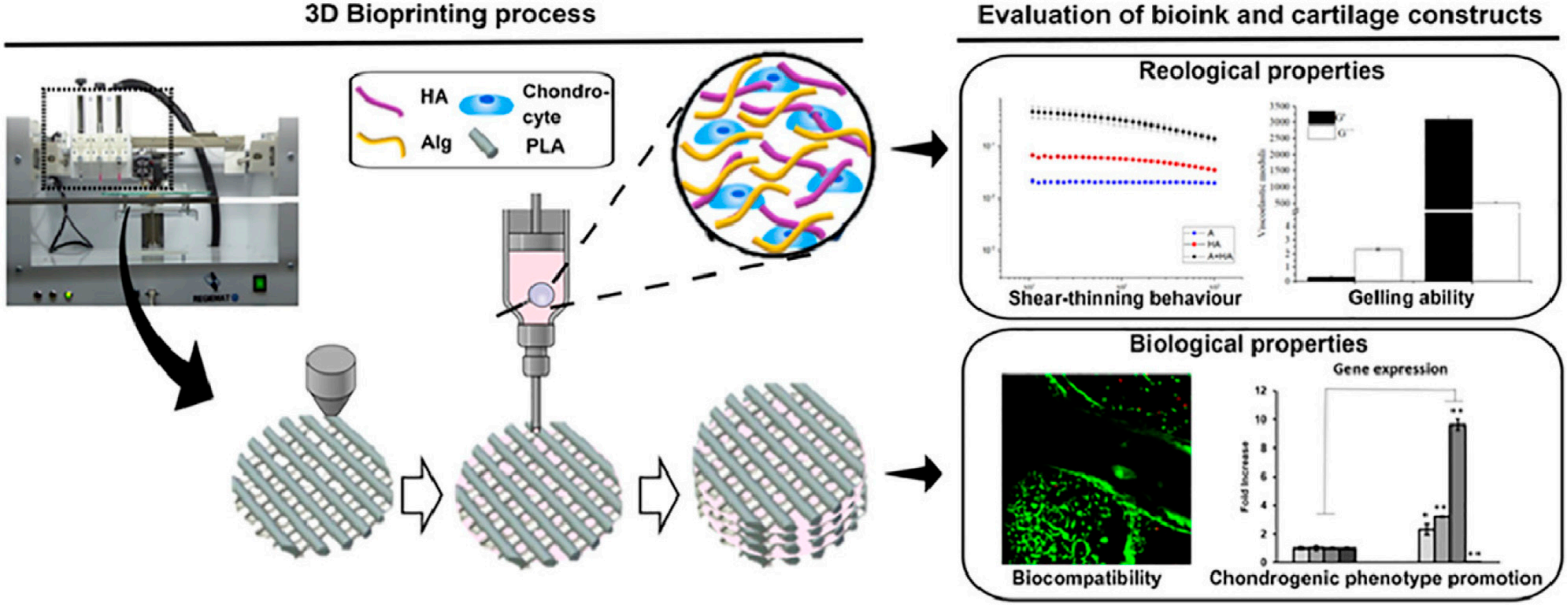
Schematic preparation of PLA-based bioprinting scaffolds of articular cartilage engineering. Reproduced from Antich et al., (2020) with permission from Copyright 2016 Elsevier.

PLA was an aliphatic polymer with high mechanics, suitable processability, and rheological properties for 3D bioprinting of high-resolution scaffolds. Antich et al. developed a versatile bio-ink comprising hyaluronic acid (HA) and PCL components *via* the 3D bioprinting technology for the cartilage repair (Figure 9) (Antich et al., 2020). This bio-ink possessed good printability, biocompatibility, and biodegradability to allow the cell viability and improve the expression of chondrogenic gene markers and specific matrix deposition, thus demonstrating excellent cartilage tissue formation. Narayanan et al. developed a fibrous hydrogel bio-ink consisting of alginate, PLA, and human adipose-derived stem cells (hASCs) (Narayanan et al., 2016). *In vitro* results showed the outstanding hASC viability and proliferation over 8 weeks, and *in vivo* experiments displayed the higher levels of cell proliferation, collagen, and proteoglycans of bioprinted meniscus for promoting chondrogenic differentiation and cartilage repair.

## Future Outlook and Concluding Remarks

Tissue engineering is an interdisciplinary field from materials engineering to life science, which aims to restore, maintain, and promote organizational functions. Based on the deep understanding of the composition, organization, and architectures, tissue engineering scaffold can be designed, constructed, and implanted into patients for achieving damaged tissue repair and regeneration. Therefore, suitable structures and appropriate functions to mimic the tissue are significantly important, wherein the cells, biomaterials, growth factors/drugs, biomechanical stimulation, and others have been systemically investigated for tissue engineering in recent years. However, scaffold-based strategies have failed to mimic the complex structure (e.g., gradient pore size and spatial heterostructures) of native tissues to place various types of cells in desirably respective positions, which cannot reiterate the complex native tissues and organs because of the heterogeneous nature of biological processes.

Hydrogels have been recognized as promising vehicles for drug carriers and tissue engineering, which could offer desirable chambers for cellular encapsulation, homogenous distribution and biomechanical support. The ideal hydrogels should possess suitable pores for cell proliferation, migration, and growth by delivering nutrients throughout the interconnected networks. Accordingly, a tunable core and porous network is essential for cartilaginous tissue engineering based on the hydrogel-based scaffolds. Fortunately, the development of bioprinting technology provided the structural fabrication and precise dimensional adjustment. Hydrogel-type bio-inks can achieve interconnected porous networks and mimicking native architectures for promoting tissue regeneration through 3D bioprinting. It is mentioned that the bio-inks should not only possess biological properties for the tissue-like microenvironments but also require structural integrity and mechanical supports during the tissue reconstruction. Besides, these bioprinting constructs should also have favorable rheological behaviors, gelation mechanism, and degradation time, and qualify spatial heterogeneity to facilitate the requirement of native tissues and organs.

Currently, the selection of bioprinting materials is mainly determined by their biocompatibility with cell growth and function, as well as their printing properties such as viscosity, extrudability, and post-press stability. Understanding the material environment is necessary to develop new types of bio-inks using the 3D bioprinting technology. One such approach is to analyze the composition and distribution of ECM proteins in acellular tissue scaffolds. Recently, advanced chemical synthesis may provide more opportunities on developing more functional adaptations, which can recover their shape, size, and function in response to external stimuli. For example, 4D printing is considered as printing of stimuli-responsive material, where their shape and or function can response to the applied external stimuli (pH, light, temperature, and magnetic).

Nowadays, the possibility of modifying the physicochemical characteristics, and rheological, mechanical, and biological properties of hydrogels has been well fulfilled by various strategies and advanced techniques. However, the technology still faces a number of obstacles and challenges that overshadow its broad-spectrum applications, such as cultivating a variety of cell types, accurate selection of materials and cells for preparation of bio-inks, and delivered cells from stable constructs with suitable mechanical and biological properties. Therefore, there are still many issues that need to be addressed in practical therapeutic applications. For example, there was almost no successful clinical trial for the application of 3D bioprinting cell-laden cartilage or bone tissue, which was attributed to animal-derived components of cell culture mediums or some biomaterials with potential immunogenicity. In addition, swelling behaviors of mot hydrogels were hard nut to crack because the swelling hydrogels were more easily destroyed during the storage, transport, or usage process, which obviously increased the harm to the body in the clinical stage. Moreover, the degradation rate of hydrogel constructs should match the regeneration speed of tissue defects, which required more reasonable design and biomaterial selection associated with the tissue bioprinting technology. Last but not least, the environmental stimulus responsiveness, self-healing properties, and multifunction should also be required for the future biomedical hydrogel-based bio-inks. Despite this development in the field of tissue engineering, including the use of hydrogels and graft-replicated ECM, many factors remain to be explored to enable tissue engineering to play a role in a wide range of biomedical and clinical applications. The properties of hydrogel-based scaffolds can be improved by combining different natural or synthetic hydrogels and using 3D-printing techniques to manufacture the structures. Based on the above considerations, scientists, engineers, and clinicians need to work more closely together to not only consult the feasibility, practicality, and multifunction of hydrogel-based bio-inks but also identify unmet requirements and prioritize their design from the bench to the clinic.
